# Critical appraisal of advance directives given by patients with fatal acute stroke: an observational cohort study

**DOI:** 10.1186/s12910-016-0166-5

**Published:** 2017-02-02

**Authors:** A. Alonso, D. Dörr, K. Szabo

**Affiliations:** 1 Department of Neurology, Medizinische Fakultät Mannheim, University of Heidelberg, Theodor-Kutzer-Ufer 1-3, 68167 Mannheim, Germany; 2 Clinical Ethics Committee, Medizinische Fakultät Mannheim, University of Heidelberg, Theodor-Kutzer-Ufer 1-3, 68167 Mannheim, Germany

**Keywords:** Advance directives, Stroke, End-of-life decision-making, Advance care planning

## Abstract

**Background:**

Advance directives (AD) imply the promise of determining future medical treatment in case of decisional incapacity. However, clinical practice increasingly indicates that standardized ADs often fail to support patients’ autonomy. To date, little data are available about the quality and impact of ADs on end-of-life decisions for incapacitated acute stroke patients.

**Methods:**

We analyzed the ADs of patients with fatal stroke, focusing on: (a) their availability and type, (b) stated circumstances to which the AD should apply, and (c) stated wishes regarding specific treatment options.

**Results:**

Between 2011 and 2014, 143 patients died during their hospitalization on our stroke unit. Forty-two of them (29.4%) had a completed and signed, written AD, as reported by their family, but only 35 ADs (24.5%) were available. The circumstances in which the AD should apply were stated by 21/35 (60%) as a “terminal condition that will cause death within a relatively short time” or an ongoing “dying process.” A retrospective review found only 16 of 35 ADs (45.7%) described circumstances that, according to the medical file, could have been considered applicable by the treating physicians. A majority of patients objected to cardiopulmonary resuscitation (22/35, 62.9%), mechanical ventilation (19/35, 54.3%), and artificial nutrition (26/35, 74.3%), while almost all (33/35, 94.3%) directed that treatment for alleviation of pain or discomfort should be provided at all times even if it could hasten death.

**Conclusions:**

The prevalence of ADs among patients who die from acute stroke is still low. A major flaw of the ADs in our cohort was their attempt to determine single medical procedures without focusing on a precise description of applicable scenarios. Therefore, less than half of the ADs were considered applicable for severe acute stroke. These findings stress the need to foster educational programs for the general public about advance care planning to facilitate the processing of timely, comprehensive, and individualized end-of-life decision-making.

## Background

In the late 1970s, advance directives (ADs) [1] were introduced in the US as means to shape end-of-life care according to individuals’ wishes and preferences. It was not until 2009, that the German Federal Parliament passed a law that regulates the requirements and legitimacy of ADs, demanding written and personally signed documents, which, in turn, are considered binding for medical decision-making [2]. Standard AD forms frequently encourage patients to determine which specific medical treatments should (not) be applied – especially life-sustaining treatments – if the patient is incapacitated in the future. However, ADs have performed poorly in the past in that only a minority of patients have completed such documents [3, 4]. The potential reasons for low completion rates include a lack of knowledge among lay people about individual medical procedures listed in ADs, apprehension that life-sustaining treatment might be stopped too soon when an AD is in place [3], and a fundamental reluctance to confront one’s owns end-of-life [5]. Considering the challenge of completing ADs, it is surprising that professional consultation by healthcare providers [3, 6] is not regularly offered to support prospective engagement in end-of life decision-making.

Even when an AD is completed, several problems with its implementation may arise. Most patients prefer to rely on standard AD forms and are reluctant to “micromanage” their own death [7], leaving much room for interpretation of the patient’s wishes by physicians and/or relatives in critical situations. Thus, several studies have found that ADs have relatively little effect on end-of-life decision-making [8, 9]. In order to improve end-of-life care, substantial efforts have been undertaken to enhance the implementation and quality of ADs in order to increase physicians’ knowledge about patients’ preferences [8, 10]. However, most studies of ADs have included patients with diseases that traditionally are the focus of palliative medicine, such as cancer, congestive heart failure, chronic obstructive pulmonary disease [8], dementia [11], or HIV/AIDS [12]. In contrast, only a few studies have evaluated the preferences for end-of-life treatments in situations such as stroke [13, 14]. This is particularly striking as stroke accounted for one of every 20 deaths in the US in 2011, leading to a total of 128,932 stroke deaths [15]. Based on data from the WHO Mortality Database, 11% of all deaths in Europe are attributable to stroke [16]. Consequently, there is an urgent need to improve individualized and timely advance care planning with special regard to end-of-life scenarios due to stroke.

In this study, we aimed to analyze ADs in dying stroke patients. We concentrated on the predefined circumstances to which the individual AD should apply and the stated choices for specific treatment options to retrospectively analyze the impact of the AD on end-of-life decisions in stroke patients.

## Methods

We identified all patients admitted with a diagnosis of ischemic stroke or spontaneous intracranial hemorrhage (ICH) who died during their hospitalization on our 29-bed comprehensive stroke unit (SU) from January 2011 to December 2014. Ischemic or hemorrhagic stroke was confirmed by CT or MRI of the brain for all the patients. Out of these, we retrospectively analyzed the medical records of those patients with advance directives. Patients with existing written ADs were included for further analyses. Patients with a remark in their charts indicating an AD existed, according to their next of kin, but whose AD was not available in the charts were excluded. Likewise, patients with a written power of attorney for healthcare without a concomitant AD were excluded. In order to estimate the prognosis in terms of early mortality risk as a basis for further decision-making, we retrospectively assessed two different well-established risk scores of each patient at the time of admission (the IScore [17] or ICH Score [18], and the Risk Score for In-Hospital Death in Patients Admitted with Ischemic or Hemorrhagic Stroke [19]).

We analyzed ADs according to the following criteria and content: (a) type of AD, (b) applicability to the circumstances at presentation, and (c) specifications referring to medical and therapeutic actions (such as diagnostic procedures, nutrition, medication, and initiation of palliative measures).

## Results

### Patients

**Fig. 1:**
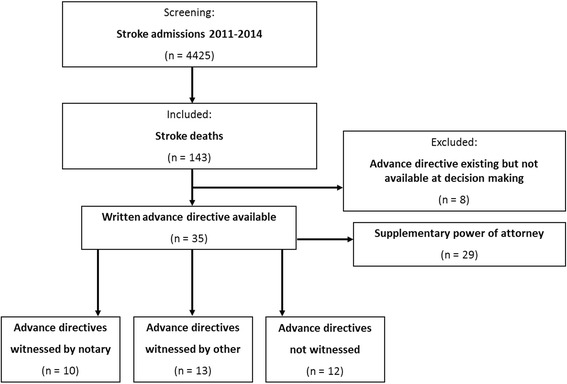
Cohort selection

Of 4425 patients admitted with either ischemic stroke or ICH, 143 patients died during the subsequent hospitalization. Of these, 42 patients (29.4%) had completed and signed a written AD; however, at the time of decision-making, only 35 ADs were available and could be examined (Fig. 1). Thirteen of these 35 (37.1%) patients were male. The mean age of all the patients was 83.3 ± 8.5 years at the time of the stroke. Twenty-nine of the 35 (82.9%) patients had been admitted with a diagnosis of ischemic stroke and 6 (17.1%) had suffered from an ICH. From a clinical perspective, most of the patients were severely affected, with a median score of 19 (range = 5–26) at admission on the National Institute of Health Stroke Scale (NIHSS). The proportion of deceased patients with signed ADs increased over the observation period, from 22.6% having an AD in 2011 to 43.6% in 2014.

**Fig. 2:**
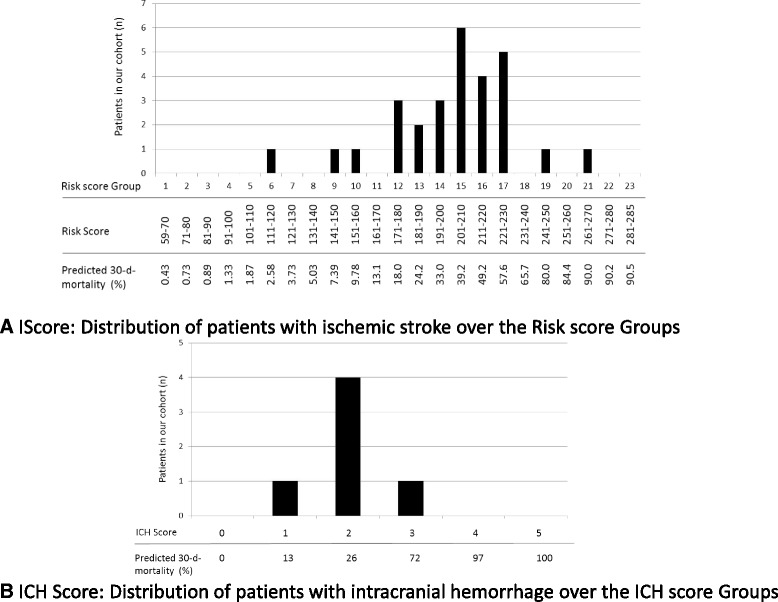
Outcome prognosis of our cohort according to the IScore for patients with ischemic stroke (**a**) and according to the ICH Score for patients with intracerebral haemorrhage (**b**)

With regard to the mortality assessment IScore [17], 7 of the 29 (24.1%) patients with ischemic stroke had a score >221, indicating a predicted 30-day mortality of 57.6%. Of these, only 1 patient had a score indicating a predicted 30-day mortality of 80 and 90%, respectively (Fig. 2a). With respect to the ICH score [18], 1 patient had a score of 3, which predicted a 30-day mortality of 72%, and no patient had a score of 4 (predicting a 30-day mortality of 97%) or 5 (predicting a 30-day mortality of 100%; Fig. 2b). Applying a categorical risk score for both ischemic and hemorrhagic stroke (a model including the NIHSS Score [19]), no patient achieved a score that was associated with a >50% risk of in-hospital mortality.

Of the 35 patients, 26 (74.3%) had at least one chronic disease which was either potentially life-limiting or predisposed them to a possible persistent inability to make decisions. The presence of single or multiple advanced organic diseases was diagnosed in 10 patients (28.6%), including end-stage renal disease with hemodialysis (3/35, 8.6%), congestive heart failure (6/35, 17.1%), and chronic obstructive lung disease (2/35, 5.7%). Eight patients (22.9%) suffered from cardiovascular disease with severe sequelae, either with a diagnosis of previous stroke (5/35, 14.3%) or prior myocardial infarction (1/35, 2.9%), or both (2/35, 5.7%). Six patients (17.1%) had a diagnosis of cancer, and nine patients suffered from a neurodegenerative disease (dementia in 6/35, 17.1%; Parkinson’s disease in 2/35, 5.7%; and both diseases in 1/35, 2.9%).

### AD forms

All ADs fulfilled the minimal legal requirements, that is: a written form that was personally signed. Twenty-three ADs were witnessed; 10 by the primary care physician, 10 by a notary, 2 by a family member, and 1 by both the primary care physician and a family member. Of the 10 physicians who signed an AD, only 4 physicians stated that the criteria of informed consent or refusal had been fulfilled. Five physicians only confirmed the decision-making capacity, and 1 physician signed the AD without making any comments. Only 2 ADs had been updated and reconfirmed; the oldest AD was 15 years old and the most recent AD had been signed 19 days prior to the stroke. Twenty-nine patients (82.9%) also had named a power of attorney for healthcare. Of the 25 patients without notarial certified ADs, all except 1 patient used standard AD forms, as provided by the German Department of Justice, medical associations, or patients’ associations.

### AD content

#### Applicability to severe stroke

At the time of presentation, all 35 patients were incapable of decision-making due to either global aphasia, disturbance of consciousness, or severe neuropsychological deficits. There was considerable variety concerning the stated circumstances in which the AD should apply (see Table 1). The most frequent was for a “*terminal condition that will cause death within a relatively short time*” or ongoing “*dying process*” which was included in 21 of the 35 ADs (60%). Other common terms included situations of “*permanent unconsciousness/irreversible coma*” (48.6%), “*irreversible loss of ability to reason/of power of judgment/of decision-making ability*” (34.3%), “*end-stage condition of an incurable/fatal disease, even if death is not yet conceivable*” (31.5%), and “*permanent brain damage*” (28.6%). About one quarter wanted the AD to apply in case of “*advanced degenerative brain disease with need for artificial nutrition*” or “*failure of vital functions*” (25.7% each). Only a minority stated that the AD should cover situations involving “*most severe physical disability/disease,*” “*intolerable pain*” (8.6% in each), “*no improvement over 3 weeks after severe stroke*” (5.7%) or “*no will to live*” (2.9%). Only 1 patient made personal amendments with regard to his chronic disease (Parkinson’s disease).

**Table 1:** Stated circumstances in which the individual advance directive should apply

Qualifying scenario	Listed in	Applicable in index event	If scenario was interpreted to include:
Terminal condition that will cause death within a relatively short time/dying process	21 (60.0%)	?	
Permanent unconsciousness/irreversible coma	17 (48.6%)	0 (0.0%)	
Irreversible loss of ability to reason/of power of judgment/of decision-making ability	12 (34.3%)	6 (50.0%)	Global aphasia
End-stage condition of an incurable/fatal disease, even if death is not yet conceivable	11 (31.4%)	?	
Permanent brain damage	10 (31.4%)	9 (90.0%)	(Sub)total MCA infarction (*n* = 5) Bilateral MCA infarction (*n* = 1) Basal ganglia hemorrhage > 50 ml (*n* = 2) Basilar artery occlusion (*n* = 1)
Advanced degenerative brain disease with need of artificial nutrition	9 (25.7%)	0 (0.0%)	
Failure of vital functions	9 (25.7%)	1 (11.1%)	Sepsis with multiple organ failure
Most severe physical disability/disease	3 (8.6%)	2 (66.7%)	Hemiplegia, bedridden
Intolerable pain	3 (8.6%)	0 (0.0%)	
No improvement over 3 weeks after severe stroke	2 (5.7%)	0 (0.0%)	
No will to live perceptible	1 (2.9%)	0 (0.0%)	

In order to appreciate patients’ wishes for end-of-life care, we retrospectively analyzed, based on the medical files, whether each AD would have been considered applicable in the course of medical treatment by employing the already mentioned model-based prediction of prognosis, combined with the clinicians’ recorded assessments. The phrasing most applicable in the presented clinical conditions was “*permanent brain damage*,” which was accurate in 9 of 10 (90%) patients. The criterion of “*most severe physical disability/disease*” and “*irreversible loss of ability to reason/of power of judgment/of decision-making ability*” was fulfilled in 2 of 3 (66.7%) and 6 of 12 (50%) patients, respectively. A “*failure of vital functions*” occurred in 1 of 9 (11.1%) patients, while none of the other statements covered the clinical situations recorded in the medical files. It was particularly challenging to evaluate situations, such as “t*erminal condition that will cause death within a relatively short time/dying process*” and the “*end-stage condition of an incurable/fatal disease, even if death is not yet conceivable.*” Based on the results of the risk-score assessment at the time of admission, a reliable prognosis of fatality could only be made during further progression of the disease, depending on clinical developments. In total, only 16 of the 35 patients (45.7%, including multiple applying assignments in 2 patients) had chosen a description of circumstances that indeed applied to the presented clinical situation.

### AD treatment specifications

There also was considerable variation in refusing specific treatments under the named clinical condition. A majority of the 35 patients objected to cardiopulmonary resuscitation (62.9%), mechanical ventilation (54.3%), and artificial nutrition (74.3%). Only a few patients explicitly stated they did not want hydration (11.4%), antibiotics (5.7%), or hemodialysis (2.9%). No patient commented on his/her preference about blood transfusions. Eight of the 35 patients refused to allow organ donation upon his/her death. Thirty-three patients (94.3%) directed that treatment for alleviation of pain or discomfort should be provided at all times even if it could hasten death.

**Fig. 3:**
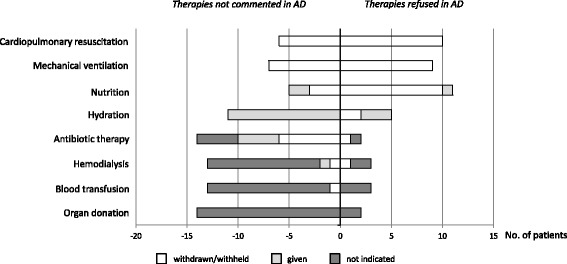
Treatment specifications of 16 patients with ADs applicable in the acute stroke setting: Number of patients who gave or withdrew/withheld specific therapies based on the AD content

Treatment specifications were quite similar when analyzing only the 16 ADs that were judged to apply to the actual clinical situation. Most of these patients objected to cardiopulmonary resuscitation (10/16), mechanical ventilation (9/16), and artificial nutrition (11/16). Yet, there were comments about hydration (5/16), antibiotic therapy (2/16), hemodialysis (3/16), blood transfusion (3/16), and organ donation (2/16). In general, refusal of specific treatments was respected by the treating physicians if the AD was found to be applicable. The only exceptions were continuation of nutrition in 1 of the 16 patients and continuation of hydration in 3 of the 16 patients, despite their explicit refusal (Fig. 3).

### Implementation of ADs

A limitation of therapeutic measures at any time point during the clinical course was documented in 34 of the 35 patients; a do-not-resuscitate order was implemented for 1 patient with referral to his AD. In 21/35 patients, an additional waiver of intensive care measures was documented in 11/20 with referral to the AD, in 9/20 after discussing the treatment limitation with the next of kin or legal guardian in order to elaborate the patient’s putative will, and in 1 patient without a documented decision-making process. Comfort care measures only were initiated for 12 of the 35 patients. This was done for 9 patients with reference to their AD, for 2 patients after discussing the treatment limitation with the next of kin, in order to elaborate on the patient’s putative will, and for 1 patient without a documented decision-making process.

## Discussion

This retrospective study explored the occurrence, content, and implications of ADs recorded in the charts of acute stroke patients who were admitted and subsequently died during hospitalization. Written ADs were found in only 35 out of 143 charts between the years 2011 and 2014. Yet, we noticed a progressive rise in ADs during the course of the observation period. Most ADs were preprinted standardized forms that contained no individualized reference to the signer or to stroke-related scenarios. Instead, the ADs mostly referred to specific stand-alone medical options and clinical states, which with lay people usually are not familiar.

### AD-related obstacles for self-determined end-of-life-care

In accordance with nationwide and international observations, our data confirmed that the elderly are increasingly attempting to take pertinent actions to determine their own end-of-life care [20, 21]. Indeed, over the 4-year-period of this study the proportion of deceased stroke-patients that had an AD nearly doubled, reaching 43.6% in 2014. In addition, 82% of the AD signers had designated a future surrogate. Yet, the findings still indicate low engagement with the topic, given the documented incremental need for autonomous end-of-life decision-making within populations in western cultures [22].

To date, consistent with our findings, most AD-holders in Germany use standard AD forms that are provided by several institutions [23]. On one hand, these forms are useful because they only require a person to choose singular stand-alone treatment options. On the other hand, standardized ADs mostly fail to reflect the complexity of specific scenarios in critical illness. Moreover, the impact of a critical illness on an individual’s life might be ever so unpredictable and complex that it cannot be judged in advance, as critics of ADs have stressed [5, 24]_._ However, some scenarios for progressive chronic diseases may be foreseeable. In our study, 26 patients (74.3%) had at least one chronic disease that was potentially life-limiting or predisposed them to future states of decisional inability. Likewise, 14.3% had an instance of stroke in their medical history. Surprisingly, only one patient made a personal amendment to the AD with special regard to his progressive (Parkinson’s) disease. Obviously, the majority of patients did not (want to) anticipate the potential course of their health conditions and correspondently did not take the best possible actions to regulate their end-of-life care. In a study by Hawkins et al., only a few individuals wished to document preferences and mandate that specific life-sustaining treatments should be followed without exception [7]. This could be one reason for the low completion rates of ADs, which press individuals to make such declarations. Yet, imprecise definitions of treatment choices are commonly regarded as a weakness of ADs. As Qureshi et al. [9] discuss, the poor impact of ADs may be due to an insufficient specification of treatments that patients can choose or refuse. In our study, patients used forms that had them comment on measures, such as antibiotic treatment or hemodialysis. A case can be made that specific therapeutic options should be left to the treating physicians, according to medical indications in relation to given contexts.

Spokoyny et al. [25] proposed a specific AD for stroke that aimed at coordinating treatment options for stroke scenarios related to time windows and evidence levels. The authors claim to support patients’ autonomy by providing adequate information that has a decisive effect on therapeutic decision-making [25]. However, the contrary may be the case: leaving evermore specific treatment decisions to non-medically trained individuals – particularly, within a highly specific and fictional medical context – may lead to overextended and burdensome advance decision-making, which may not lead to increased autonomy. One main flaw of ADs may be, in fact, their lack of reference to treatment goals, since ADs focus mainly on therapeutic measures. Several authors have concluded that the concept of ADs has failed to accomplish what they were originally conceived to do; namely, to enable individuals to determine their future medical care based on the principle of informed consent [26]. White and Arnold [27] point out that treatment-limiting ADs, in the first place, give legal (likewise psychological) permission to surrogates and doctors to stop life-support in designated circumstances after life-support already has been initiated. Yet, it is preferable to clarify if such measures should be initiated after all.

### Questioning AD quality in acute stroke context

Evaluating the quality of ADs in terms of their applicability (relevance in the presented clinical condition) and validity (phrasing that reflects the informed attitude of the signer) requires that their wording be given close attention. In our study, only 16 of 35 ADs were judged to fit the clinical assessment in our retrospective analyses. Thus, the complexity of decision-making processes could certainly not be fully reconstructed and evaluated on a retrospective basis. Decisions about life-sustaining measures for “terminal conditions” or an “ongoing dying process,” which were stated in 60% of the ADs, should be based fundamentally on arguments about medical futility rather than on the patient’s wishes. Yet, in our study it appeared to be difficult to estimate current situations as being fatal. Most obviously, pre-formulated terminology as “permanent brain damage” or “most severe disability” demand close scrutiny of the pertinent underlying concepts while attempting to convert the forwarded patient’s will. While medical professionals might focus on neuro-anatomic damages that most probably lead to severe disability, most lay people might instead bear in mind (permanent) functional losses (e. g., not being able to walk, or to attend to own bodily needs, global aphasia, etc.). Respectively, the idea of future impairment of capabilities raises fundamental fears and questions about intrinsic values in life and what is considered to be a good quality of life. The findings of Hanger et al. showed that 82% of controls preferred death to the severe disability caused by stroke [28]. Similarly, a recent study assessed healthcare workers’ opinions about acceptable stroke outcomes following decompressive hemicraniectomy (a procedure that reduces mortality rates but may lead to severe disability). Most participants stated survival with dependency to be unacceptable [29]. However, the retrospective consent rates in stroke survivors after the procedure ranged between 79 and 100% [30–32]. This emphasizes the *disability paradox,* as preferences may change over time with a decline in health and may lead to a higher acceptance of given states [33, 34]. It is undisputed that little can be known about patients’ deliberations while completing ADs if there is no (written) evidence of the underlying decision process or a link to individual health-related values. In our study, only 4 out of 10 physicians who countersigned ADs stated that the informed consent criteria had been fulfilled. Unfortunately, there is a wide range of possibilities for misinterpretation on behalf of the signers and later by their surrogates and doctors. Once again, this calls into question whether current AD practices are a satisfactory and effective tool for legitimizing (crucial) medical actions.

### Need for deliberated advance care planning for stroke-related conditions

A conclusive implementation of valuable, action-guiding ADs in stroke settings has to reflect that, in contrast to other chronic diseases, severe stroke has its unique illness trajectory with special implications [35, 36]. Stroke occurs unexpectedly and patients present at their worst, having an uncertain short-term risk of mortality or a likely long-term period of disability. Furthermore, all communication has to take place through patients’ surrogates [37]. Medical lay people should obtain adequate information about the goals, means, and limitations of ADs. The content of ADs concerning choices between specific medical procedures should be made as comprehensible as possible for lay people. It is advisable that patients’ perceived preferences should be appropriately phrased and reevaluated periodically [38]. It is essential to reassess ADs in the light of the diagnosis of a severe illness, a decline in one’s health condition, or when an incisive life-event occurs, and to document personal involvement with regard to achievable and preferable treatment goals [39]. In addition, assistance by healthcare professionals should be mandatory in the AD completion processes. Finally, it is highly recommended that patients appoint a healthcare proxy who is familiar with their health-related values. The advancement of decision-making-instruments is illustrated by the advance-care planning model proposed by den Schmitten et al. for emergency cases [40]. In a structured AD amendment patients may choose between diverse options: “*life-prolonging therapy without restrictions (A*).” “*life-prolonging therapy with the following restrictions: do not resuscitate (B1), in addition, do not intubate (B2), in addition, no intensive care treatment (B2), in addition, no hospitalization (B4*)” or “*no life-prolonging therapy at all, comfort care only (C*).” This catalogue provides a manageable size of options and serves as one component of advance care planning based on careful deliberation.

### Study’s limitations

The present study has several limitations. The sample size was small because of the low rate of completed ADs. As the study was a retrospective chart analysis, details about the interpretation of ADs that were not noted in the charts could be missing. Furthermore, only patients who died during their hospitalization were included, since we assumed that the ADs in this cohort would, in fact, be noticed and considered. This approach carried the risk of overlooking a withdrawal-of-care bias [41], and calls attention to the topic of refusal of medical treatments in circumstances of almost imminent death. However, the focus of our study was primarily to evaluate the applicability and validity of ADs in patients with fatal stroke rather than analyzing their impact on decision-making processes on a more differentiated long-term basis. Certainly, it would be valuable to analyze the ADs of all patients with severe stroke, irrespective of patients’ outcomes as the determinant variable. Further prospective long-term studies are needed to elucidate this issue.

## Conclusions

ADs should enable individuals to have their wishes respected in future treatment decisions in case of incapacity. Yet, the prevalence of ADs among patients who die from acute stroke is still low. In our cohort, less than half of the existing ADs were considered to be applicable for severe acute stroke. From the clinicians’ point of view, the use of standardized ADs is not likely to support the intended autonomy of patients. There is a need to foster educational programs for the general public about advance care planning, especially in the light of the large number of stroke-related conditions in the aging population.

## Abbreviations

ACP: Advance care planning
AD: Advance directive
CT: Computed tomography
EAPC: European association for palliative care
HIV/AIDS: Human immunodeficiency virus/acquired immune deficiency syndrome
ICH: Intracranial hemorrhage
MRI: Magnetic resonance imaging
NIHSS: National Institute of Health Stroke Scale
SU: Stroke unit
WHO: World Health Organization
